# The Keap1/Nrf2 Protein Axis Plays a Role in Osteoclast Differentiation by Regulating Intracellular Reactive Oxygen Species Signaling*

**DOI:** 10.1074/jbc.M113.478545

**Published:** 2013-06-25

**Authors:** Hiroyuki Kanzaki, Fumiaki Shinohara, Mikihito Kajiya, Tetsuya Kodama

**Affiliations:** From ‡ Maxillo-Oral Disorders,; § Department of Orthodontics, and; ¶ Oral Medicine and Surgery, Department of Oral and Maxillofacial Surgery, Tohoku University Hospital, Sendai 980-8575,; ‖ Oral Microbiology, Tohoku University Graduate School of Dentistry, Sendai 980-8575,; the ** Department of Periodontal Medicine, Division of Applied Life Science, Hiroshima University Graduate School of Biomedical and Health Sciences, Hiroshima 734-8551, and; the ‡‡ Molecular Delivery System Laboratory, Department of Biomedical Engineering, Graduate School of Biomedical Engineering, Tohoku University, Sendai 980-8575, Japan

**Keywords:** Arthritis, Bone, Nrf2, Osteoclast, Periodontal Disease, Reactive Oxygen Species (ROS)

## Abstract

Reactive oxygen species (ROS) act as intracellular signaling molecules in the regulation of receptor activator of nuclear factor-κB ligand (RANKL)-dependent osteoclast differentiation, but they also have cytotoxic effects that include peroxidation of lipids and oxidative damage to proteins and DNA. Cellular protective mechanisms against oxidative stress include transcriptional control of cytoprotective enzymes by the transcription factor, nuclear factor E2-related factor 2 (Nrf2). This study investigated the relationship between Nrf2 and osteoclastogenesis. Stimulation of osteoclast precursors (mouse primary peritoneal macrophages and RAW 264.7 cells) with RANKL resulted in the up-regulation of kelch-like ECH-associated protein 1 (Keap1), a negative regulator of Nrf2. It also decreased the Nrf2/Keap1 ratio, and it down-regulated cytoprotective enzymes (heme oxygenase-1, γ-glutamylcysteine synthetase, and glucose-6-phosphate dehydrogenase). Nrf2 overexpression up-regulated the expression of cytoprotective enzymes, decreased ROS levels, decreased the number of tartrate-resistant acid phosphatase-positive multinucleated cells, reduced marker genes for osteoclast differentiation, and attenuated bone destruction in both *in vitro* and *in vivo* models. Overexpression of Keap1 or RNAi knockdown of Nrf2 exerted the opposite actions. In addition, *in vivo* local Nrf2 overexpression attenuated lipopolysaccharide-mediated RANKL-dependent cranial bone destruction *in vivo*. This is the first study to show that the Keap1/Nrf2 axis regulates RANKL-dependent osteoclastogenesis through modulation of intracellular ROS signaling via expression of cytoprotective enzymes. This raises the exciting possibility that the Keap1-Nrf2 axis may be a therapeutic target for the treatment of bone destructive disease.

## Teaser

**Background:** Nuclear factor E2-related factor 2 (Nrf2) is a master regulator of cytoprotective enzymes.

**Results:** Nrf2 overexpression-mediated cytoprotective enzymes' augmentation blocked RANKL signaling via intracellular ROS attenuation and thereby blocked bone destruction.

**Conclusion:** Nrf2-dependent cytoprotective enzyme expressions play a role in the regulation of osteoclastogenesis by controlling intracellular ROS.

**Significance:** The Keap1/Nrf2 axis could be a novel therapeutic target for the treatment of bone destructive disease.

## Introduction

The osteoclast is a multinucleated cell that can resorb bone tissue (1) and is derived from a hematopoietic lineage (2), specifically the macrophage-monocyte cell line (3). Osteoclastogenesis is strictly regulated by receptor activator of nuclear factor-κB ligand (RANKL)^2^ (4), which is expressed on osteoclastogenesis-supporting cells such as activated lymphocytes (5), stromal cells (6), and osteoblasts (7). The currently known intracellular signaling pathways activated after receptor binding of RANKL include the nuclear factor of activated T-cells (8), mitogen-activated protein kinases (MAPKs) (9), tumor necrosis factor (TNF) receptor-associated factors (TRAFs) (10, 11), c-Jun N-terminal kinases (JNKs) (12), and reactive oxygen species (ROS) (13).

It is well known that ROS act not only as intracellular signaling molecules but also exert cytotoxic effects such as peroxidation of lipids and phospholipids (14) and oxidative damage to proteins and DNA (15). Cells have several protective mechanisms against these oxidative stressors (16). One of the major cellular antioxidant responses is the induction of cytoprotective enzymes, including antioxidative and carcinogen-detoxification enzymes; this occurs through the operation of the cytoplasmic oxidative stress system, which may be activated by a variety of natural and synthetic chemopreventive agents (17). Transcriptional factor nuclear factor E2-related factor 2 (Nrf2) transcriptionally controls the gene expression of many cytoprotective enzymes, such as heme oxygenase-1 (HO-1) (18), NAD(P)H:quinone reductase (NQO1) (19), γ-glutamylcysteine synthetase (GCS) (20), and glucose-6-phosphate dehydrogenase (21). However, kelch-like ECH-associated protein 1 (Keap1) negatively regulates Nrf2-dependent transcription of cytoprotective enzymes by inhibiting nuclear translocation of Nrf2, with cytoplasmic ubiquitination and degradation of Nrf2 (22).

Although ROS have been reported to act as intracellular signaling molecules to regulate osteoclast differentiation, little is known about the cytoprotective mechanisms against oxidative stress during osteoclastogenesis, particularly the regulation of cytoprotective enzyme expression by the Keap1/Nrf2 axis. In this study, we hypothesized that Nrf2-dependent induction of cytoprotective mechanisms against oxidative stress would be attenuated during RANKL-mediated osteoclastogenesis. To investigate this hypothesis, an *in vitro* cell culture system and an *in vivo* bone destruction model system were utilized.

## EXPERIMENTAL PROCEDURES

### 

#### 

##### Animals

All experimental protocols were approved by the Internal Animal Care and Use Committee, Tohoku University, Japan.

##### Mouse Peritoneal Macrophages

Three C57/B6 mice (Japan SLC Inc., Hamamatsu, Japan) were sacrificed, and cold phosphate-buffered saline solution (PBS) was injected into the peritoneal cavities. Peritoneal lavage fluid was collected and centrifuged to harvest the cells. The cells recovered from the precipitated pellet were pre-cultured with 50 ng/ml of recombinant macrophage colony-stimulating factor (Wako Pure Chemical Industries, Ltd., Osaka, Japan) for 4 days, and the attached cells were used as peritoneal macrophages (23).

##### Mouse Cells from RAW 264.7 Monocytic Cell Line

RAW 264.7 cells were obtained from the Riken BioResource Center (Tsukuba, Japan).

##### Cell Culture

Mouse peritoneal macrophages and RAW 264.7 cells were cultured in α-modified Eagle's medium (Wako Pure Chemical Industries, Ltd.) containing 10% (v/v) fetal bovine serum (HyClone; Thermo Scientific, South Logan, UT) supplemented with antibiotics (100 units/ml penicillin and 100 μg/ml streptomycin). All cells were cultured at 37 °C in a 5% CO_2_ incubator.

##### In Silico Gene Chip Analysis of Mouse Osteoclastogenesis

The publicly available microarray dataset for mouse osteoclastogenesis was downloaded from the Genome Network Platform. Cells from the mouse macrophage cell line, RAW 264.7, were stimulated with 100 ng/ml RANKL for 1 day, and the gene expressions at day 0 and day 1 were compared. Using this dataset, the expressions of Keap1, Nrf2 and cytoprotective enzymes were analyzed.

##### RNA Interference

Validated siRNAs specific for mouse Nrf2 were purchased from Santa Cruz Biotechnology, Inc. (Santa Cruz, CA). Nrf2-specific siRNAs or nonsilencing siRNAs were transfected into RAW 264.7 cells using the X-tremeGENE siRNA transfection reagent (Roche Applied Science). siRNA (100 nmol/liter final concentration) was diluted into dilution medium (without serum, antibiotic-free) in 6-well plates; X-tremeGENE siRNA reagent was added, and this was incubated for 20 min at room temperature. A suspension of RAW 264.7 cells (50 × 10^4^ cells/well) was then added, and the cells cultured for 24 h with medium containing the transfection reagent.

##### Overexpression of Keap1 and Nrf2

Expression plasmids encoding human KEAP1 (hrGFP-Keap1; Addgene plasmid 28025) (24) and human NRF2 (pcDNA3-EGFP-C4-Nrf2; Addgene plasmid 21549) (25) were obtained, respectively, from Dr. Qing Zhong (University of California, Berkeley, CA) and Dr. Yue Xiong (University of North Carolina, Chapel Hill) via Addgene. The expression plasmids were transfected into RAW 264.7 cells using the X-tremeGENE HP DNA transfection reagent (Roche Applied Science). After culture for 24 h with medium containing the transfection reagent, the cells were used to confirm overexpression of Keap1 or Nrf2, or for the osteoclastogenesis assay.

##### Intracellular ROS Detection

Intracellular ROS levels were detected using the Total ROS/Superoxide Detection Kit (Enzo Life Sciences Inc., Farmingdale, NY), according to the manufacturer's instructions. Cells were stimulated with soluble RANKL (sRANKL; Wako Pure Chemical Industries, Ltd.) for 6 h, washed, and collected in PBS supplemented with 2% fetal bovine serum (FBS). The cell suspension was incubated with oxidative stress detection reagent for 30 min on ice. After washing with PBS, intracellular ROS were detected using an Accuri C6 flow cytometer (BD Biosciences). The viable monocyte/macrophage fraction was gated on an FSC/SSC plot, and ROS levels were monitored in the FL-1 channel.

##### Osteoclastogenesis Assay

Cells were plated onto 96-well plates at a density of 10^3^ cells/well (RAW 264.7 cells) or 10^4^ cells/well (mouse primary peritoneal macrophages), in the presence or absence of recombinant sRANKL (50 ng/ml final concentration). The mouse primary peritoneal macrophage culture was supplemented with recombinant macrophage colony-stimulating factor (20 ng/ml). After culture, cells were stained for tartrate-resistant acid phosphatase (TRAP) using an acid phosphatase kit (Sigma) according to the manufacturer's instructions. Dark-red multinucleated cells (≥3 nuclei) were counted as TRAP-positive multinucleated cells.

##### Resorption Assay

Mouse primary peritoneal macrophages were seeded on synthesized calcium phosphate substrate (bone resorption assay plate; PG Research, Tokyo, Japan) and stimulated with RANKL. After 7 days of cultivation, cells were removed with bleach, and the calcium phosphate substrate was washed with distilled water and then dried. Photographs were taken, and the average of the resorbed area per field was calculated from 12 images of each sample, using ImageJ software (National Institutes of Health, Bethesda).

##### Real Time RT-PCR Analysis of the Expression of Keap1/Nrf2 Genes and Osteoclast Differentiation Marker Genes

RNA was extracted from cultured cells using the GenElute mammalian total RNA miniprep kit (Sigma) according to the manufacturer's instructions. After measurement of the RNA concentration, isolated RNA (100 ng each) was reverse-transcribed with iScript cDNA Supermix (Bio-Rad), and cDNA stock was diluted (5×) with Tris/EDTA (TE) buffer. Real time RT-PCR was performed with SsoFast EvaGreen Supermix (Bio-Rad) using a CFX96 instrument (Bio-Rad). The PCR primers used in the experiments were from Primerbank and are described in Table 1. We chose six representative marker genes (ATP6v0d2 (26), cathepsin K (27), dendritic cell-specific transmembrane protein (DC-STAMP) (28), matrix metallopeptidase-9 (MMP9) (29), osteoclast-associated receptor (OSCAR) (30), and TRAP (31) for osteoclast differentiation.

**TABLE 1:** **The PCR primers used in the experiments**

Gene (gene ID)	Direction	Sequence	PrimerBank ID/Ref.
Mouse *Rps18* (6222)	Up	AGTTCCAGCACATTTTGCGAG	6755368a1
	Down	TCATCCTCCGTGAGTTCTCCA	
Mouse *Keap1* (50868)	Up	TGCCCCTGTGGTCAAAGTG	7710044a1
	Down	GGTTCGGTTACCGTCCTGC	
Mouse *Nrf2* (18024)	Up	GCCCACATTCCCAAACAAGAT	6754832a2
	Down	CCAGAGAGCTATTGAGGGACTG	
Mouse *HO-1* (15368)	Up	AAGCCGAGAATGCTGAGTTCA	6754212a1
	Down	GCCGTGTAGATATGGTACAAGGA	
Mouse *NQO1* (18104)	Up	AGGATGGGAGGTACTCGAATC	6679080a1
	Down	AGGCGTCCTTCCTTATATGCTA	
Mouse *GCS* (14629)	Up	GGGGTGACGAGGTGGAGTA	33468897a1
	Down	GTTGGGGTTTGTCCTCTCCC	
Human *KEAP1* (9817)	Up	CTGGAGGATCATACCAAGCAGG	45269144b1
	Down	GAACATGGCCTTGAAGACAGG	
Human *NRF2* (4780)	Up	TTCCCGGTCACATCGAGAG	224028256b3
	Down	GCATACCGTCTAAATCAACAGGG	
Mouse *MMP9* (17395)	Up	TCCAGTACCAAGACAAAG	44
	Down	TTGCACTGCACGGTTGAA	
Mouse *TRAP* (11433)	Up	TACCTGTGTGGACATGACC	44
	Down	CAGATCCATAGTGAAACCGC	
Mouse *Cathepsin K* (13038)	Up	TGTATAACGCCACGGCAAA	44
	Down	GGTTCACATTATCACGGTCACA	
Mouse *OSCAR* (232790)	Up	CTGCTGGTAACGGATCAGCTCCCCAGA	45
	Down	CCAAGGAGCCAGAACCTTCGAAACT	
Mouse *Atp6v0d2* (242341)	Up	GAAGCTGTCAACATTGCAGA	26
	Down	TCACCGTGATCCTTGCAGAAT	
Mouse *DC-STAMP*(75766)	Up	TGGAAGTTCACTTGAAACTACGTG	45
	Down	CTCGGTTTCCCGTCAGCCTCTCTC	
Mouse *RANKL* (21943)	Up	CCAAGATCTCTAACATGACG	46
	Down	CACCATCAGCTGAAGATAGT	
Mouse *TNF-*α (21926)	Up	CCCTCACACTCAGATCATCTTCT	7305585a1
	Down	GCTACGACGTGGGCTACAG	

##### Preparation of Nuclear Protein Lysate

Nuclear protein lysate was prepared from RAW 264.7 cells using the DUALXtract cytoplasmic and nuclear protein extraction kit (DualSystems Biotech AG, Schlieren, Switzerland) according to the manufacturer's instructions. Cultured cells were washed with PBS and treated with cell lysis buffer. After centrifugation, the nuclear pellet was washed twice and lysed with nuclear lysis reagent. After centrifugation, the cleared supernatant was used as the nuclear protein extract. The protein concentrations of each of the nuclear lysates were measured with the Quick Start^TM^ protein assay kit (Bio-Rad), and the concentrations were adjusted to be the same. After mixing with 4× sample buffer containing β-mercaptoethanol, the samples were heat-denatured at 70 °C for 10 min.

##### Western Blot Analysis

The prepared nuclear lysates, containing equal amounts of protein, were electrophoresed on a TGX Precast gel (Bio-Rad), and the proteins were transferred to a polyvinylidene difluoride (PVDF) membrane using an iBlot blotting system (Invitrogen). The transferred membrane was treated with Qentix Western blot signal enhancer (Thermo Fisher Scientific Inc., Rockford, IL) according to the manufacturer's instructions. After washing with deionized water, the membrane was blocked with BlockAce (DS Pharma Biomedical Inc., Osaka, Japan) for 30 min, and then incubated for 1 h with rabbit IgG anti-Nrf2 antibody (Santa Cruz Biotechnology) in Can Get Signal Solution-1 (Toyobo Co. Ltd., Tokyo, Japan). After thorough washing with PBS containing 0.5% Tween 20 (PBS-T), the membrane was incubated for 1 h with HRP-conjugated protein A/G (Thermo Fisher Scientific Inc.) in Can Get Signal Solution-2 (Toyobo Co. Ltd) and washed with PBS-T. Chemiluminescence was produced using Luminata Forte (EMD Millipore Corp., Billerica, MA) and detected with an ImageQuant LAS-4000 digital imaging system (GE Healthcare). To confirm the equivalence of loaded nuclear protein, the membrane was re-probed with Restore Plus Western blot stripping buffer (Thermo Fisher Scientific Inc.) for 15 min, washed, blocked, and then blotted in anti-histone H3 antibody (Cell Signaling Technology Japan, Tokyo, Japan) followed by HRP-conjugated protein A/G.

Cytoplasmic protein samples were used for the detection of HO-1, NQO1, and GCS. The antibodies used in these experiments were anti HO-1 antibody (StressMarq Biosciences Inc., Victoria, British Columbia, Canada), anti-NQO1 antibody (Abcam plc, Cambridge, MA), and anti-GCS antibody (Thermo Fisher Scientific Inc.). To confirm the equivalence of loaded cytoplasmic protein, TGX Stain-Free precast gels (Bio-Rad) were used for cytoplasmic protein samples. Proteins in the electrophoresed gel were visualized with ultraviolet (UV) treatment before transfer to a PVDF membrane. Visualized protein on the membrane was imaged with an ImageQuant LAS-4000 under UV transillumination, and the value of the total protein band density was used for calibration.

##### In Vivo Bone Destruction Model

We utilized repeat injections of LPS for our *in vivo* bone destruction model (32). This bone destruction model induces RANKL-dependent osteoclastogenesis and extensive bone destruction. Thirty two 8-week-old C57B/6 male mice were used in the experiments. The mice were randomized into four equal groups as follows: mock vector injection group (control group); local Nrf2 overexpression group (Nrf2 overexpression group); LPS-induced bone resorption group (LPS group); and LPS-induced bone resorption and local Nrf2 overexpression group (LPS + Nrf2 overexpression group). Purified LPS from *Escherichia coli* O111:B4 (Sigma) was injected at a dose of 10 μg per site, with transfection reagent, on days 1, 3, 5, 7, and 9. For *in vivo* transfection, an HVJ envelope-vector kit (GenomONE, Ishihara-Sangyo Kaisha Ltd., Osaka, Japan) was used, according to the manufacturer's instructions (33). Administration of the HVJ envelope vector containing the human NRF2 expression plasmid (8 μg) was performed at the same time and sites as LPS or PBS injection. Nrf2 vector solution (containing human NRF2 expression plasmid enveloped by HVJ) was injected under anesthesia with a 30-gauge needle at a point on the midline of the skull located between the eyes on days 1, 3, 5, 7, and 9. Mock vector (the original expression plasmid pcDNA3) was injected into the corresponding area as a control in the same way.

On day 11, the mice were sacrificed by cervical dislocation. RNA from the excised cranial tissue around the injected site (three RNA samples per group) was extracted and reverse-transcribed as detailed above. Gene expression was examined by real time PCR. Other cranial tissue samples (five samples per group) were fixed overnight with 4% paraformaldehyde in PBS, and the specimen was subsequently scanned with an x-ray microtomography (microCT) system (ScanXmate-E090; Comscantecno Co., Ltd., Kanagawa, Japan). The scanned microCT images were reconstituted using ConeCTexpress software (Comscantecno Co., Ltd.). After reconstitution and obtaining the DICOM files, three-dimensional volume rendering was performed using Pluto software.

##### Statistical Analysis

All data are presented as the mean ± S.D. All *in vitro* data are the means of three independent experiments. Multiple comparisons were performed with Tukey's test. *p* < 0.05 was considered to be statistically significant.

## RESULTS

### 

#### 

##### In Silico Gene Chip Analysis of Mouse Osteoclastogenesis

Microarray analysis using publicly available dataset revealed that both Keap1 and Nrf2 were down-regulated following stimulation of cells with RANKL (Table 2). The Nrf2/Keap1 ratio in RANKL-stimulated RAW 264.7 cells was significantly lower than that in control RAW 264.7 cells. To determine whether Nrf2-dependent cytoprotective enzyme expression had been reduced, the expression of cytoprotective enzymes was analyzed, and it was found that all cytoprotective enzymes examined (HO-1, GCS, glucose-6-phosphate dehydrogenase, and peroxiredoxin) were down-regulated following stimulation with RANKL. These results suggest that stimulation with RANKL favors intracellular ROS signaling by attenuating the expression of cytoprotective enzymes.

**TABLE 2:** **Microarray analysis of mouse osteoclastogenesis**

Gene	Probe ID	Signal intensity		Fold change from day 0
		Day 0	Day 1	
*Nrf2*	1416543_at	868.2	645.8	0.744
*Keap1*	1450746_at	190.0	168.4	0.886
			Nrf2/Keap1 fold change ratio	0.840
*HO-1*	1448239_at	2653.1	296.9	0.112
*GCS*	1424296_at	820.9	289.1	0.352
*G6PD*	1422327_s_at	1095.7	575.4	0.525
Peroxiredoxin (*Prdx*)*-1*	1433866_x_at	2061.9	1239.2	0.601
*Prdx-5*	1416381_a_at	949.4	542.2	0.571

##### Nrf2/Keap1 Ratio and Expression of Cytoprotective Enzymes Were Decreased in sRANKL-stimulated RAW 264.7 Cells

**FIGURE 1.**
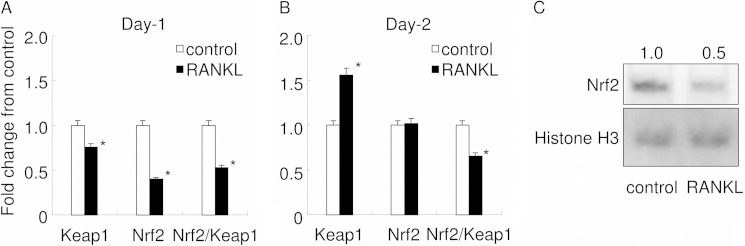
**Keap1 and Nrf2 expression in RANKL-stimulated RAW 264.7 cells.** The expressions of Keap1 and Nrf2 and the Nrf2/Keap1 ratio were examined by real time PCR at day 1 (*A*) and day 2 (*B*). Gene expression was calibrated using the RPS18 housekeeping gene, and values indicating the fold-change from control are shown. *Open bars* represent controls, and *closed bars* represent stimulation by RANKL. *C* shows nuclear translocation of Nrf2 in RAW 264.7 cells. Equal amounts of nuclear protein extracted from control or RANKL-stimulated RAW 264.7 cells were electrophoresed and transferred to PVDF membrane. The membrane was subjected to Western blot analysis for Nrf2 and histone H3. Densitometry analysis was used to calculate the ratio relative to the control, and the mean values of three experiments are shown. The data shown are representative of three independent experiments performed in triplicate. *, *p* < 0.05 *versus* control.

Next, it was confirmed whether the expressions of Nrf2 and Keap1 were changed during RANKL-mediated osteoclastogenesis by real time PCR (Fig. 1, *A* and *B*). At day 1, the same time point as that used for the microarray analysis, the expressions of both Keap1 and Nrf2 were down-regulated. Of note, Nrf2 was extensively down-regulated compared with Keap1, resulting in a reduced Nrf2/Keap1 ratio. At day 2, Keap1 expression was up-regulated, maintaining the decrease in the Nrf2/Keap1 ratio, although the reduction seemed attenuating at day 2. Fig. 1*C* shows Western blot analysis of intranuclear level of Nrf2. RANKL stimulation gave reduction of intranuclear level of Nrf2. These results suggest that Nrf2-dependent cytoprotective enzyme expression may be attenuated not only at day 1 but also at day 2, which would favor intracellular ROS signaling.

**FIGURE 2.**
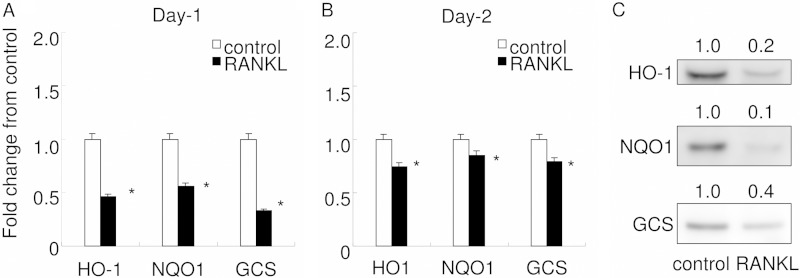
**Cytoprotective enzymes are down-regulated by RANKL stimulation.** The expressions of HO-1, NQO1, and GCS were examined by real time PCR at day 1 (*A*) and day 2 (*B*). Gene expression was calibrated using the RPS18 housekeeping gene, and values indicating the fold-change from control are shown. *Open bars* represent controls, and *closed bars* indicate stimulation by RANKL. The data shown are representative of three independent experiments performed in triplicate. *, *p* < 0.05 *versus* control. *C* shows Western blot analysis for HO-1, NQO1, and GCS in RAW 264.7 cells. Equal amounts of cytoplasmic protein lysate were electrophoresed and transferred to PVDF membrane. To confirm the equivalence of loaded protein, TGX Stain-Free Precast Gels were used. Proteins in the electrophoresed gel were visualized with UV treatment before transfer to a PVDF membrane. Visualized protein on the membrane was imaged under UV transillumination, and the value of the total band density was used for calibration. Densitometry analysis was used to calculate the ratio relative to the control, and the mean values of three experiments are shown *above* each panel.

To determine whether this might be the case, the expression of cytoprotective enzymes was measured in RANKL-stimulated RAW 264.7 cells in mRNA level (Fig. 2, *A* and *B*) and protein level (Fig. 2*C*). All cytoprotective enzymes assessed (HO-1, NQO1, and GCS) were down-regulated by stimulation with RANKL, both at day 1 and day 2 (Fig. 2, *A* and *B*). As expected, protein level expressions of HO-1, NQO1, and GCS were all attenuated. These results suggest that stimulation with RANKL favors intracellular ROS signaling by attenuating the expression of cytoprotective enzymes.

##### Keap1 or Nrf2 Overexpression, or Nrf2 Knockdown, in RAW 264.7 Cells

**FIGURE 3.**
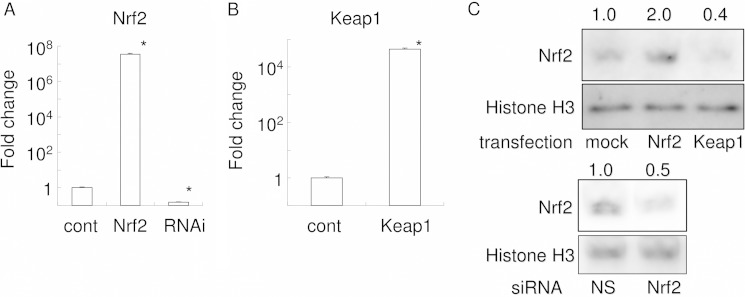
**Nuclear translocation of Nrf2 is increased by Nrf2 overexpression and decreased by Keap1 overexpression or Nrf2 knockdown, altering the transcription of cytoprotective enzymes.** The expressions of Nrf2 (*A*) and Keap1 (*B*) were examined by real time PCR at day 1. Gene expression was calibrated using the RPS18 housekeeping gene, and values indicating the fold-change from control are shown. *A,* compares expression levels for human Nrf2 between control (*cont*), transfection with human Nrf2 (up-regulation), and transfection with Nrf2 RNAi (knockdown); *B,* compares expression levels for human Keap1 between control and transfection with human Keap1 (up-regulation). The data represent the means of three independent experiments performed in triplicate. *, *p* < 0.05 *versus* control. *C,* nuclear translocation of Nrf2. Equal amounts of nuclear protein were electrophoresed and transferred to PVDF membrane. The membrane was subjected to Western blot analysis for Nrf2 and histone H3. The *upper panels* show the comparison between mock and Nrf2 transfection, and the *lower panels* show the comparison between nonsilencing and Nrf2 siRNA. Densitometry analysis was used to calculate the ratio relative to the control, and the mean values of three experiments are shown for Nrf2, *above* each respective panel. *NS,* no significant difference between groups.

To further clarify the role of the Keap1/Nrf2 axis in RANKL-mediated osteoclastogenesis, expression plasmids for Keap1 or Nrf2, or siRNA for Nrf2, were transfected into RAW 264.7 cells, and real time PCR analysis was performed at day 1 (Fig. 3, *A* and *B*). Transfection of the Nrf2 or Keap1 expression plasmids successfully induced high expressions of human Nrf2 mRNA or Keap1 mRNA, respectively, in RAW 264.7 cells. Transfection of siRNA for Nrf2 reduced Nrf2 mRNA expression. The effects of overexpression/knockdown were observed even at day 4 (data not shown). Western blot analysis for Nrf2 using nuclear protein extracts revealed an increase in nuclear Nrf2 in Nrf2-transfected cells (Fig. 3*C*). In contrast, both Keap1 overexpression and Nrf2 RNAi decreased nuclear Nrf2. These data thus verify that our transfection and RNAi techniques had successfully modified intranuclear level of Nrf2.

##### Cytoprotective Enzyme Expression in RAW 264.7 Cells Was Increased by Nrf2 Overexpression but Decreased by Keap1 Overexpression and Nrf2 Knockdown

**FIGURE 4.**
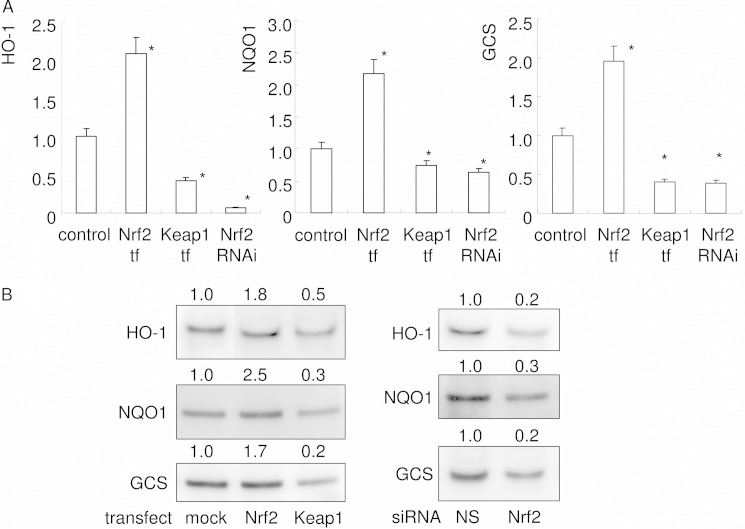
**Expression of cytoprotective genes was up-regulated by Nrf2 overexpression and down-regulated by Keap1 overexpression or Nrf2 knockdown.** The expressions of HO-1, NQO1, and GCS were examined by real time PCR at day 2 (*A*). Gene expression was calibrated using the Rps18 housekeeping gene, and values indicating the fold-change from control are shown. The data represent the means of three independent experiments performed in triplicate. *, *p* < 0.05 *versus* control. *B,* Western blot analysis for HO-1, NQO1, and GCS. Cytoplasmic extracts containing equal amounts of protein were electrophoresed and transferred to PVDF membrane. The membrane was subjected to Western blot analysis. To confirm the equivalence of loaded cytoplasmic protein, TGX Stain-Free Precast Gels were used. Proteins in the electrophoresed gel were visualized with UV treatment before transfer to a PVDF membrane. Visualized protein on the membrane was imaged under UV transillumination, and the value of the total band density was used for calibration. Densitometry analysis was used to calculate the ratio relative to the control, and the mean values of three experiments are shown *above* each panel. *NS,* no significant difference between groups.

Next, real time PCR was used to observe the effects of changes in intranuclear level of Nrf2 on the expression of cytoprotective enzymes (Fig. 4*A*). A decrease in Nrf2 nuclear translocation, induced by Keap1 overexpression or Nrf2 RNAi, was associated with a significant reduction in the mRNA expressions of HO-1, NQO1, and GCS. In contrast, the enhancement of Nrf2 nuclear translocation induced by Nrf2 overexpression significantly increased the mRNA expression of these cytoprotective enzymes. These results indicate that our transfection and RNAi techniques were able to regulate transcriptionally the Nrf2-dependent expression of cytoprotective enzymes. Western blot analysis revealed that protein level expressions of HO-1, NQO1, and GCS were augmented by Nrf2 overexpression (Fig. 4*B*). In contrast, protein level expressions of HO-1, NQO1, and GCS were attenuated by Keap1 overexpression and Nrf2 knockdown. These data thus verify that our transfection and RNAi techniques had successfully modified Nrf2-dependent cytoprotective enzyme expression.

##### Intracellular ROS Levels in sRANKL-stimulated RAW 264.7 Cells Were Decreased by Nrf2 Overexpression and Increased by Keap1 Overexpression or Nrf2 Knockdown

**FIGURE 5.**
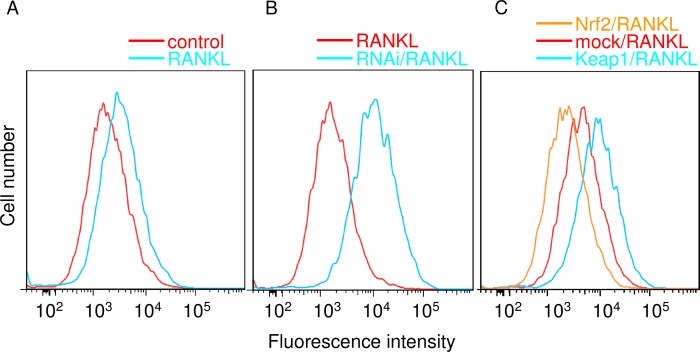
**Detection of intracellular ROS.** Control RAW cells and cells with overexpression/knockdown of Keap1/Nrf2 were stimulated with sRANKL for 6 h, and the ROS levels were examined using flow cytometry. *A,* RANKL induced intracellular ROS. The *red line* indicates the control, and the *cyan line* indicates stimulation with RANKL. The data shown are representative of three independent experiments performed in triplicate. *B,* Nrf2 knockdown increased intracellular ROS levels in RANKL-stimulated RAW cells. The *red line* indicates RANKL stimulation of cells transfected with nonsilencing siRNA, and the *cyan line* indicates RANKL stimulation of cells transfected with Nrf2 siRNA. The data shown are representative of three independent experiments performed in triplicate. *C,* intracellular ROS levels in RANKL-stimulated RAW cells were decreased by Nrf2 overexpression and increased by Keap1 overexpression. The *red line* indicates RANKL stimulation of cells transfected with mock vector; the *cyan line* indicates RANKL stimulation of cells transfected with the Keap1 expression plasmid, and the *orange line* indicates RANKL stimulation of cells transfected with the Nrf2 expression plasmid. The data shown are representative of three independent experiments performed in triplicate.

Flow cytometry was used to determine the effects of exogenous changes in cytoprotective enzyme expression on intracellular ROS levels in RAW 264.7 cells (Fig. 5). Stimulation with RANKL substantially increased intracellular ROS levels in RAW 264.7 cells (Fig. 5*A*). Nrf2 RNAi, shown above to decrease cytoprotective enzyme expression, further increased intracellular ROS levels as compared with RANKL stimulation alone (Fig. 5*B*). Similarly, Keap1 overexpression also elevated intracellular ROS levels (*cyan line* in Fig. 5*C*). In contrast, Nrf2 overexpression, shown above to increase cytoprotective enzyme expression, reduced intracellular ROS levels as compared with RANKL stimulation alone (*orange line* in Fig. 5*C*). These results suggest that RANKL-mediated ROS production is regulated by the Keap1/Nrf2 axis via the expression of cytoprotective enzymes.

##### Osteoclastogenesis Was Reduced by Nrf2 Overexpression and Increased by Keap1 Overexpression or Nrf2 Knockdown

**FIGURE 6.**
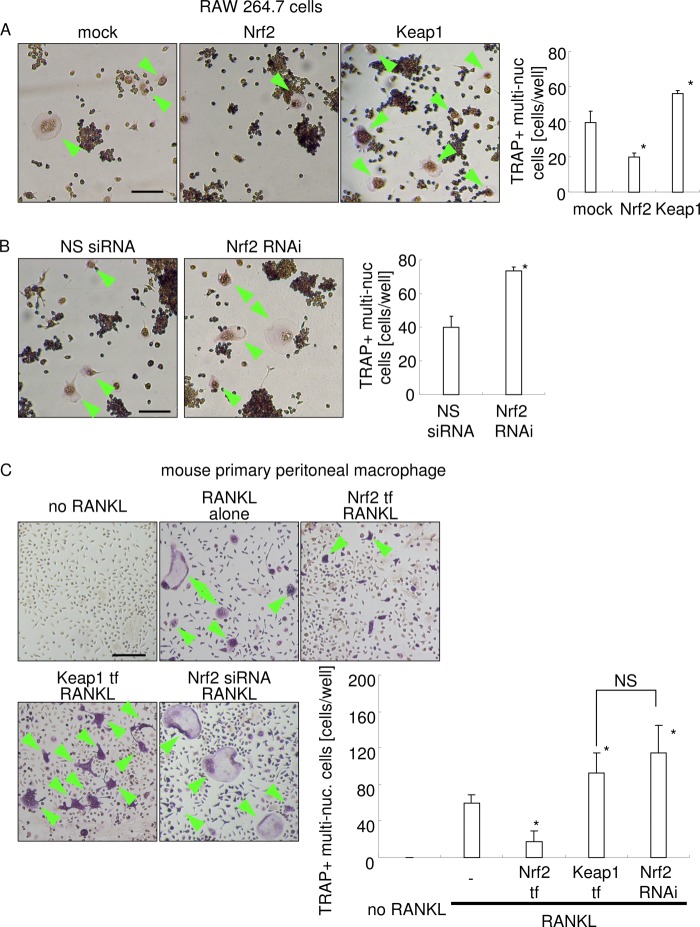
**Osteoclastogenesis assay.** *A,* mock, Nrf2, and Keap1 expression plasmids were transfected into RAW 264.7 cells; the cells were then stimulated with RANKL for 3 days, and TRAP staining was performed. The data shown are representative of three independent experiments performed in triplicate. *, *p* < 0.05 *versus* mock transfection. *Bar,* 100 μm. *Arrowheads* indicate TRAP^+^ multinucleated cells. *B,* nonsilencing or Nrf2 siRNA was transfected into RAW 264.7 cells; the cells were then stimulated with RANKL for 3 days, and TRAP staining was performed. The data shown are representative of three independent experiments performed in triplicate. *, *p* < 0.05 *versus* nonsilencing siRNA transfection. *Bar,* 100 μm. *Arrowheads* indicate TRAP^+^ multinucleated cells. *C,* osteoclastogenesis assay using mouse primary peritoneal macrophages. The cells were transfected with the Nrf2 or Keap1 expression plasmid, or Nrf2 siRNA, 12 h before stimulation with RANKL. The cells were stimulated with RANKL for 7 days, and TRAP staining was performed. The culture medium was exchanged once at day 4. All culture media for peritoneal macrophage culture were supplemented with recombinant macrophage colony-stimulating factor (20 ng/ml). The data shown are representative of three independent experiments performed in triplicate. *Bar,* 100 μm. *, *p* < 0.05 *versus* stimulation with RANKL alone. *NS,* no significant difference between groups.

Next, it was determined whether changes in intracellular ROS levels influenced RANKL-mediated osteoclastogenesis in the mouse macrophage cell line (RAW 264.7 cells) and mouse primary macrophage (Fig. 6). Similar results were found both in RAW 264.7 cells and in primary peritoneal macrophages. The enhancement of ROS levels by Nrf2 RNAi or Keap1 overexpression significantly increased the number of tartrate-resistant acid phosphatase-positive (TRAP^+^) multinucleated cells, compared with RANKL stimulation alone. In contrast, a reduction in ROS levels by Nrf2 overexpression significantly reduced the number of TRAP^+^ multinucleated cells, compared with RANKL stimulation alone. Not only the number of TRAP^+^ multinucleated cells but also the size of TRAP^+^ multinucleated cells were affected by knockdown or transfection of Nrf2 and Keap1.

**FIGURE 7.**
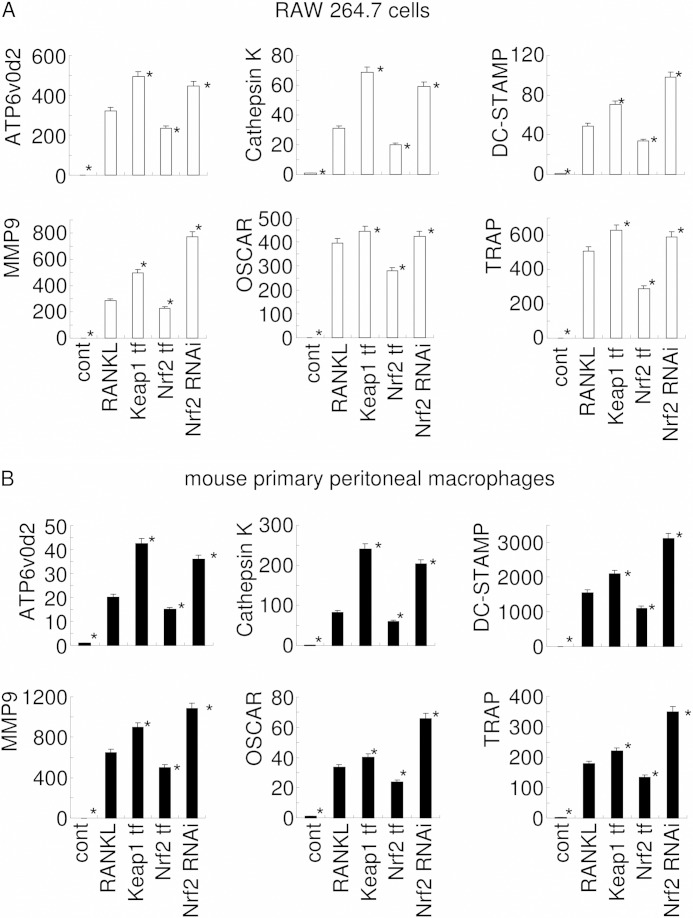
**Real time PCR analysis of osteoclast marker gene expression.** The expressions of osteoclast marker genes (ATP6v0d2, cathepsin K, DC-STAMP, MMP9, OSCAR, and TRAP) in RANKL-stimulated RAW 264.7 cells (day 3) or mouse primary peritoneal macrophages (day 7) were examined by real time PCR. Gene expression was calibrated using the Rps18 housekeeping gene, and values indicating the fold-change from control (*cont*) are shown. The data shown are representative of three independent experiments performed in triplicate. *, *p* < 0.05 *versus* stimulation with RANKL alone.

To explore further the influence of changes in intracellular ROS levels on RANKL-mediated osteoclastogenesis, the expression of osteoclast differentiation marker genes (ATP6v0d2, cathepsin K, DC-STAMP, MMP9, OSCAR, and TRAP) was examined using real time PCR (Fig. 7). Consistent with the observed changes in the numbers of TRAP^+^ multinucleated cells, the elevation of ROS levels by Nrf2 RNAi or Keap1 overexpression significantly induced the expression of osteoclast differentiation marker genes, whereas a reduction in ROS levels induced by Nrf2 overexpression was associated with a significant reduction in the expression of these genes. These data support the proposal that intracellular ROS levels, regulated by the Keap1/Nrf2 axis, play a role in osteoclastogenesis.

##### Osteoclast Resorption Activity Was Attenuated by Nrf2 Overexpression

**FIGURE 8.**
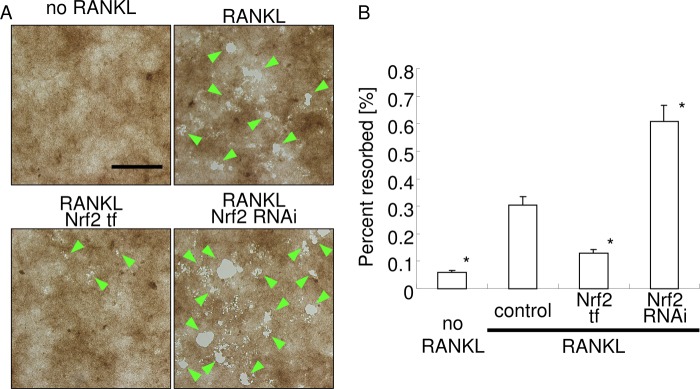
**Resorption assay.** Mouse primary peritoneal macrophages were performed Nrf2 transfection or RNAi knockdown and stimulated with RANKL. The photographs shown are representative of three experiments (*A*). *Arrowheads* indicate resorbed lacunae on substrate. *Bar,* 100 μm. Mean percent resorbed area was calculated from randomly selected six images using ImageJ software (*B*). *, *p* < 0.05 *versus* stimulation with RANKL.

Next, the effects of Nrf2 overexpression and knockdown on the resorption activity of osteoclasts were investigated (Fig. 8). Consistent with our observations of the number of TRAP^+^ multinucleated cells and the expression of osteoclast marker genes, Nrf2 overexpression caused a reduction in the resorbed area, whereas Nrf2 knockdown resulted in an increase in the resorbed area. These data indicate that exogenous regulation of Nrf2 in osteoclasts is a significant mechanism to control not only osteoclastogenesis but also osteoclast activity.

##### Local Nrf2 Overexpression Ameliorates Bone Destruction in Vivo

**FIGURE 9.**
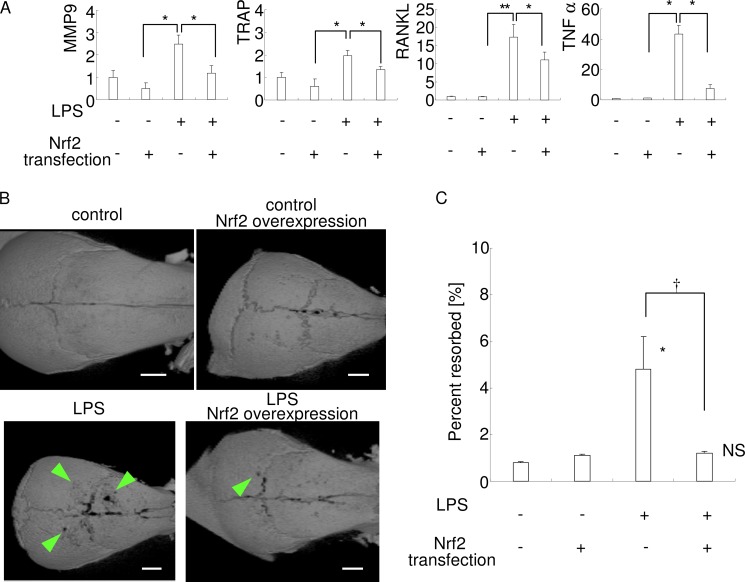
**Local Nrf2 overexpression ameliorates bone destruction *in vivo*.** LPS (10 μg/site) or PBS were injected five times into each mouse, every other day, with or without local human Nrf2 gene transfer. *A,* real time PCR analysis was performed using three RNA samples per group. Mean *C_t_* values of human Nrf2 in each group are given under “Results.” *, *p* < 0.05 between groups. *B,* x-ray microtomographic images were taken using five samples per group, and representative photographs are shown. *Arrowheads* indicate resorbed lacunae or holes formed in the cranial bone. *Bar,* 1 mm. *C,* resorbed area in the cranial bone was calculated using ImageJ software. Mean values are shown for percent resorbed (calculated from the ratio of the number of pixels in the resorbed area in the cranial bone to the number of pixels in the cranial bone in the analyzed image). *, *p* < 0.05 *versus* control. *NS,* no significant difference *versus* control. †, *p* < 0.05 between groups.

Finally, we examined whether local Nrf2 overexpression was able to reduce bone destruction *in vivo*. Local injections of lipopolysaccharide (LPS; repeated on five occasions) induced the gene expression of osteoclast markers (MMP9 and TRAP) and osteoclastogenic cytokines (RANKL and TNF-α) (Fig. 9*A*). Consistent with this induction, cranial bone resorption was observed in the samples following injection of LPS alone (Fig. 9, *B* and *C*). Real time PCR analysis for human Nrf2 revealed that local Nrf2 gene transfer gave extensive induction of human Nrf2 mRNA expression in sites (*C_t_* value are as follows: not detectable (over 50 cycles) in control or LPS injection group; 24.1 in control + Nrf2 overexpression group; and 20.8 in LPS injection + Nrf2 overexpression group, respectively). Local Nrf2 overexpression with LPS inhibited the LPS-induced gene expression of these osteoclast markers and osteoclastogenic cytokines (Fig. 9*A*), as well as LPS-induced bone resorption (Fig. 9, *B* and *C*). No statistically significant difference in bone destruction was found between the control and LPS + Nrf2 overexpression group, signifying almost complete amelioration of bone destruction was obtained by local Nrf2 overexpression. These results suggest that exogenous Nrf2 induction may be a potential therapeutic target for the treatment of bone destructive disease.

## DISCUSSION

The main findings of this study are that the Keap1/Nrf2 axis plays a major role in the regulation of RANKL-mediated osteoclastogenesis, by controlling intracellular ROS levels via transcriptional regulation of cytoprotective enzyme expression. The results, together with the publicly available microarray data, indicate that there was a reduction in the Nrf2/Keap1 ratio during osteoclastogenesis, and this led to transcriptional down-regulation of cytoprotective enzymes, signifying the induction of intracellular ROS. Our studies to detect ROS by flow cytometry, as well as those of other research groups (13, 34, 35), show an enhancement of intracellular ROS levels during RANKL-mediated osteoclastogenesis. Furthermore, our experiments involving overexpression and knockdown of Keap1 and Nrf2 clearly demonstrate that an increase in Nrf2 attenuated osteoclastogenesis, whereas a reduction in Nrf2 induced osteoclastogenesis. In addition, intracellular ROS removal by ROS scavenger such as catechin also attenuated RANKL-mediated osteoclastogenesis (data not shown). Taken together, the results provide strong evidence that the Keap1/Nrf2 axis plays a role in osteoclastogenesis by modulating intracellular ROS levels via cytoprotective enzymes.

The role of ROS in intracellular signaling has been investigated previously, revealing signal transduction between RANK and ROS. Wang *et al.* (36) reported that Rac1 is responsible for regulating ROS generation during osteoclast differentiation, and Lee *et al.* (34) reported that TRAF6 plays a key linkage role in ROS production by RANKL. Furthermore, the latter group and others have reported that ROS derived from NADPH oxidases (NOXs) may be crucial for RANKL-induced osteoclast differentiation (34, 37, 38). Integrating these data suggests that the signaling cascade from RANK to ROS production is as follows: RANK, TRAF6, Rac1, and then NADPH oxidases.

Cells have protective mechanisms against oxidative stressors such as ROS, via induction of cytoprotective enzymes through transcriptive regulation by Nrf2 (16). These protective mechanisms could potentially interfere with ROS induction by RANKL. However, to date there have been no reports, to our knowledge, concerning the cytoprotective mechanisms against oxidative stress during osteoclastogenesis, especially the regulation of cytoprotective enzyme expression by the Keap1/Nrf2 axis. In this study, it was hypothesized that Nrf2-mediated induction of cytoprotective mechanisms against oxidative stress would be attenuated during RANKL-dependent osteoclastogenesis. As predicted by this hypothesis, the Nrf2/Keap1 ratio decreased following stimulation by RANKL, and this was associated with a reduction in cytoprotective enzyme expression. The mechanism by which stimulation with RANKL reduces Nrf2 is not currently known. There is a report that Keap1 has highly reactive thiol groups in its structure and that oxidation of this domain leads to substantial conformational changes in Keap1, resulting in dissociation from Nrf2 and hence nuclear translocation of Nrf2 (39). In addition, Nrf2 appears to autoregulate its own expression through an antioxidant response element-like element located in the proximal region of its promoter (40). Taken together, this evidence implies that an increase in ROS levels induced by stimulation with RANKL may up-regulate Nrf2, although any potential increase in Nrf2 expression in osteoclast precursors following stimulation with RANKL was not detected in the present experiments. It has also been reported that Nrf2 regulates Keap1 by controlling its transcription (41). Change of stability of Nrf2 mRNA or decrease of translation by miRNA or other hidden mechanism regulates RANKL-dependent Nrf2 down-regulation. Bach1, an inhibitor of Nrf2 binding to the antioxidant-response element, could participate this mechanism, because Bach1 knock-out mice show attenuated osteoclastogenesis (42). Further extensive investigations will be required to clarify the regulatory mechanisms linking Nrf2 to stimulation with RANKL.

Induction of intranuclear Nrf2 induces up-regulation of cytoprotective enzyme, including HO-1. HO-1 catalyzes heme and produces biliverdin, iron, and carbon monoxide, which have anti-oxidative functions (43). We presumed that up-regulated cytoprotective enzyme decreases intracellular ROS, thereby attenuating ROS-mediated RANKL signaling.

In addition to the findings in cultured osteoclasts from the RAW 264.7 cell line or mouse primary peritoneal macrophages, it was also observed that local Nrf2 gene transfer caused *in vivo* inhibition of LPS-mediated RANKL-dependent osteoclastogenesis. It is likely that this phenomenon was due to direct inhibition of osteoclastogenesis by Nrf2 overexpression. However, other cells such as immune cells and osteoblast/stromal cells also participate in the regulation of osteoclastogenesis during *in vivo* bone destruction. Further investigations of the effects of Nrf2 overexpression on immune cells and osteoblast/stromal cells will be required.

**FIGURE 10.**
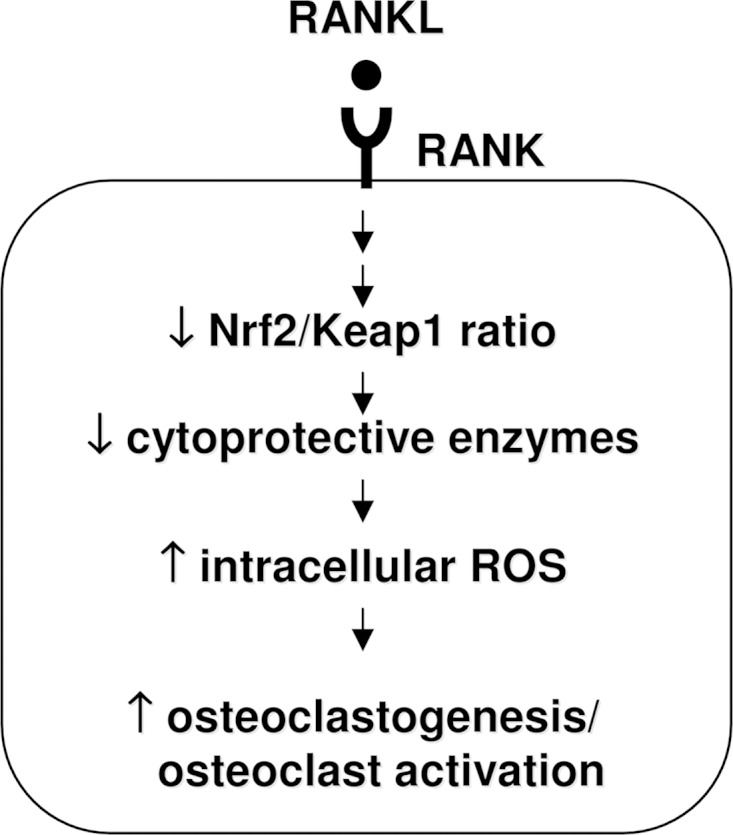
**Schematic illustration of the proposed mechanism by which the Keap1/Nrf2 axis regulates osteoclastogenesis.** First, RANKL signaling attenuates the Nrf2/Keap1 ratio, which weakens the transcription of Nrf2-dependent cytoprotective enzymes. Second, diminished levels of cytoprotective enzymes cause augmented intracellular ROS levels. Finally, osteoclastogenesis and osteoclast activation are induced.

Fig. 10 summarizes our proposed mechanism by which the Keap1/Nrf2 axis regulates osteoclastogenesis. Briefly, stimulation by RANKL reduces Nrf2 nuclear translocation, which leads to the attenuated expression of cytoprotective genes. Intracellular ROS, which are important signaling molecules downstream of RANK, may be increased by the attenuation of cytoprotective enzyme expression, thereby favoring osteoclastogenesis. Our results suggest that Nrf2 overexpression might be a potential therapeutic target for the treatment of bone destructive disease such as periodontitis and rheumatoid arthritis.

## Footnotes

2 The abbreviations used are:

RANKL: receptor activator of nuclear factor-κB ligand
RANK: receptor activator of nuclear factor-κB
DC-STAMP: dendritic cell-specific transmembrane protein
GCS: γ-glutamylcysteine synthetase
HO-1: heme oxygenase-1
Keap1: kelch-like ECH-associated protein 1
MP: matrix metallopeptidase-9
NQO1: NAD(P)H:quinone reductase
Nrf2: nuclear factor E2-related factor 2
ROS: reactive oxygen species
TRAF: TNF receptor-associated factor
TRAP: tartrate-resistant acid phosphatase
sRANKL: soluble RANKL.
